# Effects of cannabidiol on AMPKα2 /HIF-1α/BNIP3/NIX signaling pathway in skeletal muscle injury

**DOI:** 10.3389/fphar.2024.1450513

**Published:** 2024-10-22

**Authors:** Zhiquan Hou, Zhifang Wang, Jun Zhang, Yunen Liu, Zhonghua Luo

**Affiliations:** ^1^ Shenyang Medical College, Shenyang, China; ^2^ College of physical education, Yanshan University, Qinhuangdao, China

**Keywords:** cannabidiol, exercise-induced skeletal muscle injury, transcriptome sequencing, oxidative stress, inflammation

## Abstract

Cannabidiol: (CBD) is a non-psychoactive natural active ingredient from cannabis plant, which has many pharmacological effects, including neuroprotection, antiemetic, anti-inflammatory and anti-skeletal muscle injury. However, the mechanism of its effect on skeletal muscle injury still needs further research. In order to seek a scientifically effective way to combat skeletal muscle injury during exercise, we used healthy SD rats to establish an exercise-induced skeletal muscle injury model by treadmill training, and systematically investigated the effects and mechanisms of CBD, a natural compound in the traditional Chinese medicine Cannabis sativa L., on combating skeletal muscle injury during exercise. CBD effectively improved the fracture of skeletal muscle tissue and reduced the degree of inflammatory cell infiltration. Biochemical indexes such as CK, T, Cor, LDH, SOD, MDA, and GSH-Px in serum of rats returned to normal. Combining transcriptome and network analysis results, CBD may play a protective role in exercise-induced skeletal muscle injury through HIF-1 signaling pathway. The experimental results implied that CBD could down-regulate the expression of IL-6, NF-κB, TNF-α, Keap1, AMPKα2, HIF-1α, BNIP3 and NIX, and raised the protein expression of IL-10, Nrf2 and HO-1. These results indicate that the protective effect of CBD on exercise-induced skeletal muscle injury may be related to the inhibition of oxidative stress and inflammation, thus inhibiting skeletal muscle injury through AMPKα2/HIF-1α/BNIP3/NIX signal pathways.

## 1 Introduction

In recent years, the impact of exercise on health has become more and more clear, it has attracted increasing attention in maintaining good health and delaying various chronic diseases, such as coronary heart disease, diabetes, mental health (Mcleod et al., 2024; Philippot et al., 2022). However, inappropriate exercise load and excessive training can lead to skeletal muscle injury. Studies have displayed that exhaustive exercise can cause structural and functional damage to skeletal muscle (Si et al., 2024). Researches have discovered that oxidative stress and inflammation can also occur when skeletal muscle is damaged. The activities of superoxide dismutase (SOD) and glutathione peroxidase (GSH-Px) were reduced, while the interleukin (IL)-6, IL-1β, tumor necrosis factor-α (TNF-α) and reactive oxygen species (ROS) were markedly increased (Brett et al., 2020; Roy and Kumar, 2022). At present, some achievements have been made in the research of comprehensive therapies such as sports, medicine and rehabilitation, but there is no way to completely eliminate symptoms (Ishøi et al., 2020). Therefore, clarifying the mechanism of skeletal muscle injury will help to effectively prevent, treat and develop new targeted drugs, thus providing more therapeutic approaches.

In the means of drug recovery, many western drugs can eliminate fatigue and improve physical fitness, but most of them have certain toxic and side effects, and have not been further promoted and applied. Traditional Chinese medicine has been widely used over recent years. Therefore, it is urgent to develop scientific and effective Chinese medicine ingredients to improve skeletal muscle injury. *Cannabis sativa*, a traditional Chinese medicine, also known as mountain silk seedling, thread hemp, flax, wild hemp, fire hemp, is an annual herb of Moraceae (Wang et al., 2023). It is mainly cultivated in southwest, south and northeast of China. *Cannabis sativa* is often used as medicine with seeds, which is called “*Semen Cannabis*” or “*Fructus Cannabis*.” It is recorded in “The Secret of Outer Taiwan” that hemp seed can treat joint pain and muscle anxiety. Its main components include Δ9-tetrahydrocannabinol, cannabinol and *cannabidiol* (CBD) (Arkell, et al., 2020; Eisenstein, 2019; Wang et al., 2023). CBD is a non-physiologically dependent component that does not have the addictive properties of marijuana and does not downregulate cannabinoid receptors. It is widely used in clinic, including anti-depression, antipsychotic, antioxidant, analgesic, neuroprotective and adjuvant drugs for patients with advanced tumors (Dawidowicz et al., 2021; Kitrytė et al., 2018). At present, GW Pharmaceuticals and other companies have several cannabinoid products with THC and/or CBD as the main active ingredients listed in European and American countries or in the stage of research (Mouhamed et al., 2018).

Previous studies of our group have shown that CBD has certain anti-exercise myocardial injury effect in animal experiments (Zhang et al., 2022). However, there are few reports about the anti-exercise-induced muscle damage effect of CBD. Transcriptomics and network analysis were used in this study. The rat model of exhaustive exercise was established by using the treadmill method. The systematic experimental study of CBD against skeletal muscle injury was carried out using the research methods and experimental techniques of sports training, physiology, pathology and molecular biology. This study can provide scientific basis for its clinical application and further experimental research.

## 2 Materials and methods

### 2.1 Drugs and reagents

CBD, white powder, 95% purity, provided by Shenyang Institute of Metal Research, Chinese Academy of Sciences. The mammalian protein extraction kit and cytoplasmic protein preparation kit, BCA protein concentration determination Kit (BCA reagent, copper sulfate solution, protein standard, protein standard preparation), protease inhibitor and PBS crystals were provided by Hangzhou Ford Biotechnology Co., LTD. Sodium dodecyl sulfonate (SDS), Twin-20 and Twin-80 were purchased from Beijing Solebo Technology Co., LTD. Pre-staining protein Marker, SDS-PAGE gel kit (4% and 10% respectively), the ECL chemiluminescence solution, ammonium persulfate (APS) and glycine were obtained from Bio-RAD, USA. TEMED was provided by Beijing Dingguo Changsheng Biotechnology Co., LTD. Methanol, chloral hydrate, anhydrous ethanol and neutral gum were purchased from Sinopharm Chemical Reagent Co., LTD. The high protein skim high calcium milk powder was obtained from Inner Mongolia Yili Industrial Group Co., LTD. HRP labeled goat anti-rabbit secondary antibody, Keap1, Nrf2, HO-1 antibody, IL-6, IL10, TNF-α, NF-κB, AMPKα2, HIF-1α, BNIP3, and NIX antibody were obtained by American Abcam company. The GAPDH, HRP labeled goat anti-mouse secondary antibody and fluorescent secondary antibody sheep anti-rabbit were purchased from American Abcam company. The HE staining kits were obtained from Zhuhai Besso Biotechnology Co., LTD.

### 2.2 Animals

The SD male rat, aged 6–8 weeks and weighing 190–210 g, were purchased from Liaoning Changsheng Biotechnology Co., Ltd. (license No.: scxk (Liao) 2015-0001). The feeding environment of rats was 12 h of light and dark alternation, temperature and humidity can be controlled, and the standard feed can be taken at will. All animals were fed adaptively for 3 days before modeling. All participants have completed and passed the standardized training of animal experiment and feeding. All this experimental steps were in conformity to the guideline of experimental animal care and use of Shenyang Medicl University, the ethical approval number was SYYXY2022102501.

### 2.3 Establishment of skeletal muscle injury model

The rats were randomly divided into 4 groups: control group (n = 6), model group (n = 6), CBD low-dose group (50 mg/kg, n = 6) and CBD high-dose group (100 mg/kg, n = 6). The time of intragastric administration of CBD solution was 30 min before exercise. The model group was given the same amount of normal saline. The drug was administered for 7 days.

The improved Bedford incremental load exercise scheme was adopted to establish the sports injury model (Song et al., 2021). Before the experiment, rats were trained in adaptive treadmill running for 3 days, and acclimated for 30 min. The slope of the running platform was always 0°. Then seven consecutive days of exhaustion exercise were performed. The initial speed was 10 m/min, the exercise time was continued for 15 min, and the speed was increased once every 2 min, then the growth rate was 2 m/min. The speed reached 15 m/min for 15 min, the speed was increased once every 2 min, and the growth rate was 2 m/min. When the speed was 20 m/min, the speed was not increased until exhaustion. The exhaustion standard of rats is that they can’t maintain the predetermined running speed, stay on the runway baffle, and the use of light, electricity and sound stimulation to drive them away is still ineffective, accompanied by shortness of breath, prone running on the platform, and drooping head.

### 2.4 Sample collection

After the last training, the rats were weighed and given peritoneal anesthesia with 2% pentobarbital sodium. Blood was collected from the abdominal aorta and serum was separated. The gastrocnemius tissue was separated in ice bath, washed with normal saline and dried by filter paper, labeled and packaged. One part was fixed in 10% formaldehyde solution for pathological detection, and the other part was frozen in liquid nitrogen and stored in the refrigerator at −80°C for protein and genetic testing.

### 2.5 Detection of biochemical indexes

The separated serum was used to test the creatine kinase (CK), testosterone (T), corticosterone (Cor) and Lactate dehydrogenase (LDH) with the kit (Shanghai Jianglai Biotechnology Co., Ltd., China), the experimental procedures were carried out according to the kit instructions. The levels of superoxide dismutase (SOD), malondialdehyde (MDA) and glutathione peroxidase (GSH-Px) in the serum were determined according to the instructions of SOD, MDA and GSH-Px kits (Shanghai Jianglai Biotechnology Co., Ltd., China). The specific steps were summarized as follows. The Elisa kit was moved from the refrigerator at 4°C to the room temperature at 20°C for 60 min, while the serum was removed from the refrigerator for rewarming. The concentrated washing liquid was diluted and thoroughly mixed on the rotor and magnetic stirrer for use. The sample and antibody working solution are added. The sample plate was washed and the substrate was added. The end solution was added, and the OD value was detected by enzyme marker (Megu Molecular Instruments Co., LTD., CMax Plus).

### 2.6 HE staining to observe pathological changes

The gastrocnemius tissue of rats was fixed in 4% paraformaldehyde for 24 h, then dehydrated by gradient ethanol and made transparent by xylene. After being embedded in paraffin, it was sliced with a slicer (SLEE medical GmbH, B170071) with a thickness of 3-5 εm, after being baked, it was dewaxed by xylene. After rehydration with gradient ethanol, it was stained with hematoxylin-eosin and sealed with neutral gum. The pathological changes of gastrocnemius were observed under optical microscope and the images were collected.

### 2.7 Transcriptome sequencing

The RNA extraction, database construction and transcriptome sequencing of mouse skeletal muscle tissue were carried out by Suzhou Jinweizhi Biotechnology Co., Ltd. The differentially expressed genes between the control group and the model group were analyzed by DESeq2 1.16.1 software, and the obtained *p*-value was adjusted by Benjamini&Hochberg method to control the error detection rate, and the differential expression genes (DEGs) were screened on the condition that the corrected *p* < 0.05. The GO function and KEGG pathway enrichment analysis of differential genes were achieved through the ClusterProfiler package of R software. The results of GO enrichment include: biological process (BP), cellular compo⁃nent (CC) and molecular function (MF).

### 2.8 Network analysis method

#### 2.8.1 Screening of common targets between CBD and exercise-induced skeletal muscle injury

The smiles number of CBD was retrieved using pubchem database, which was imported into the Swiss Target Prediction database, and the species was set as “*Homo sapiens*” to retrieve the relevant targets of CBD. The GeneCards database was applied to screen targets related to sports skeletal muscle injury with “Sports skeletal muscle injury” as the key word and “Score>5” as the condition. The selected CBD target was mapped to the disease target, and the cross target of CBD for the treatment of exercise-induced skeletal muscle injury was obtained.

#### 2.8.2 Construction of “PPI” networks among common targets

The intersection targets of CBD and disease were imported into the srting database (https://cn.string-db.org/). The protein interaction composite score >0.15 was used as a screening condition, and the species of “*H. sapiens*” was defined, so as to obtain the target interaction network and obtain the PPI relationship. The tsv format of the network was imported into the Cytoscape software for network data analysis and beautification.

#### 2.8.3 Target pathway analysis

The intersection target of CBD and disease was input into the R language for GO biological function enrichment analysis and KEGG pathway enrichment analysis. The paths with *p* < 0.05 were selected and visualized using R language mapping.

#### 2.8.4 Construction of “component-target-pathway-disease” network

From the results of KEGG enrichment analysis, 15 target pathways with low *p*-value were selected to construct a “component-target-pathway-disease” network with common targets using the Cytoscape software, and the network analysis was conducted.

### 2.9 Real-time fluorescence quantitative PCR

Total RNA was extracted by Trizol method, its concentration and purity were detected, and 1 μg RNA was reverse-transcribed into cDNA, which was used as the template for fluorescence quantitative PCR reaction. The GAPDH was used as an internal reference, and the relative expression of RNA was calculated by 2^-ΔΔCT^ method. The specific primers are shown in Supplementary Table S1.

### 2.10 Western blotting

The 100 mg of frozen gastrocnemius tissues were added into PIPA lysis buffer, homogenized, centrifuged at 12,000 rpm for 30 min (4°C), and supernatant was taken. The BCA protein detection kit was used to detect the protein concentration. The protein was isolated by SDS-PAGE gel electrophoresis and transferred to PVDF membrane. The PVDF membrane containing protein was incubated in the closed solution for 2 h. The PVDF membranes were incubated in primary antibody, including Keap1, Nrf2, HO-1, IL-6, IL10, TNF-α, NF-κB, AMPKα2, HIF-1α, BNIP3 and NIX, and incubated overnight at 4°C. The PBST solution was washed and incubated with the corresponding secondary antibody, then slowly shaken at room temperature for 2 h. The PBST solution was used to clean the secondary antibody, PBST was washed three times at room temperature for 20 min each time. The contact non-destructive quantitative image (Ibt Life Sciences Shanghai Co., LTD., Touch Lmager XLi) was turned on for visual analysis of the protein, the ECL luminescent solution (keep in dark place) was prepared in equal proportions, the luminescent solution was added, and the front side was attached to the luminescent table. The moderate exposure time was selected and the image was saved.

### 2.11 Statistical analysis

The experimental data in this study were statistically analyzed by SPSS 26.0 software, expressed by mean ± standard deviation, and statistically analyzed by one-way analysis of variance (ANOVA). When *p* < 0.05, the difference was statistically significant. The statistical charts were obtained by Graphpad Prism 7.0.0 software.

## 3 Results

### 3.1 Effect of CBD on body weight of rats with exercise-induced skeletal muscle injury

**FIGURE 1:**
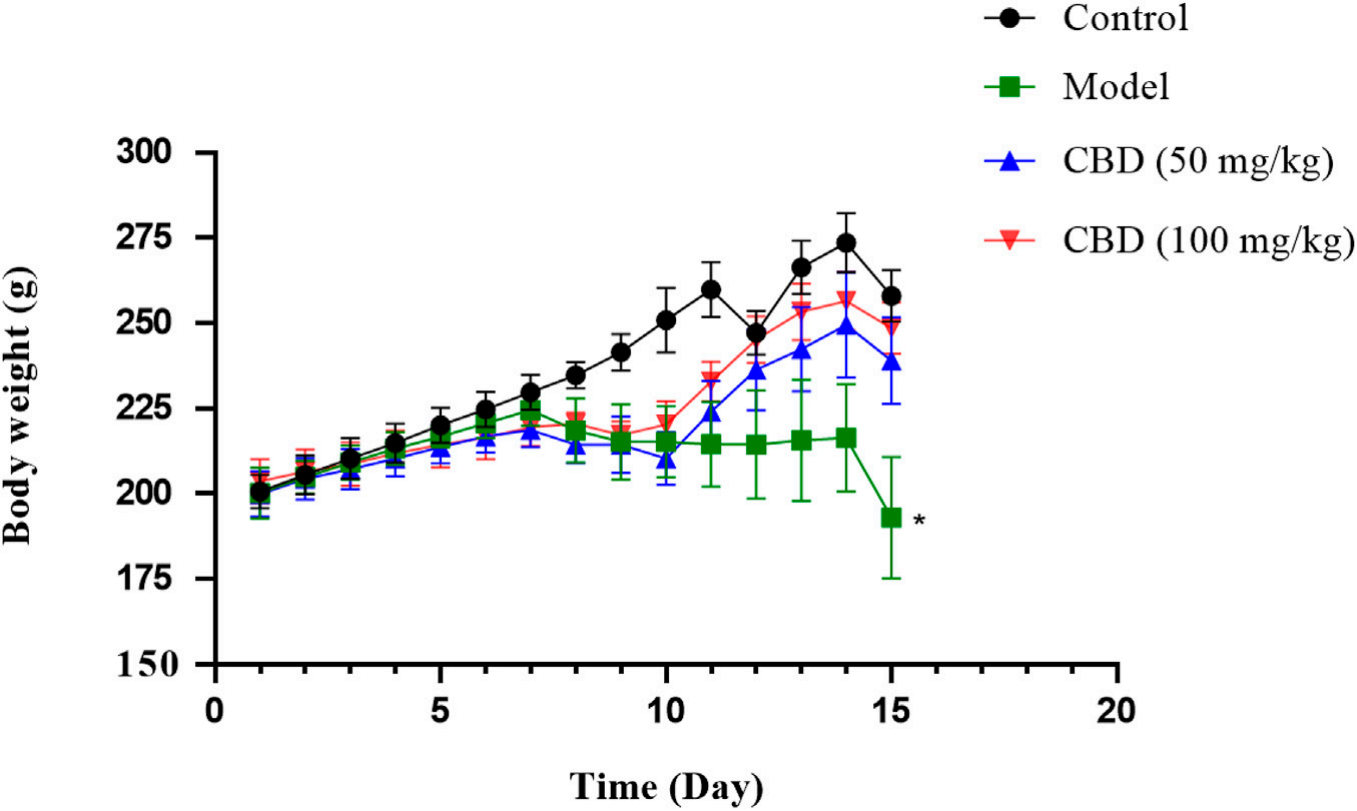
Effects of CBD body weight of experimental mice. Data are expressed as the mean ± standard deviation (SD) (n = 6, one-way ANOVA), *p < 0.05 compared with the control group.

As shown in Figure 1, after treadmill exercise, compared with the control group, the body weight of rats in the model group was decreased significantly (*p* < 0.05). Compared with the model group, the body weight of rats in the CBD dose group was increased.

### 3.2 Effect of CBD on CK, T, Cor and LDH parameters in experimental rats

**FIGURE 2:**
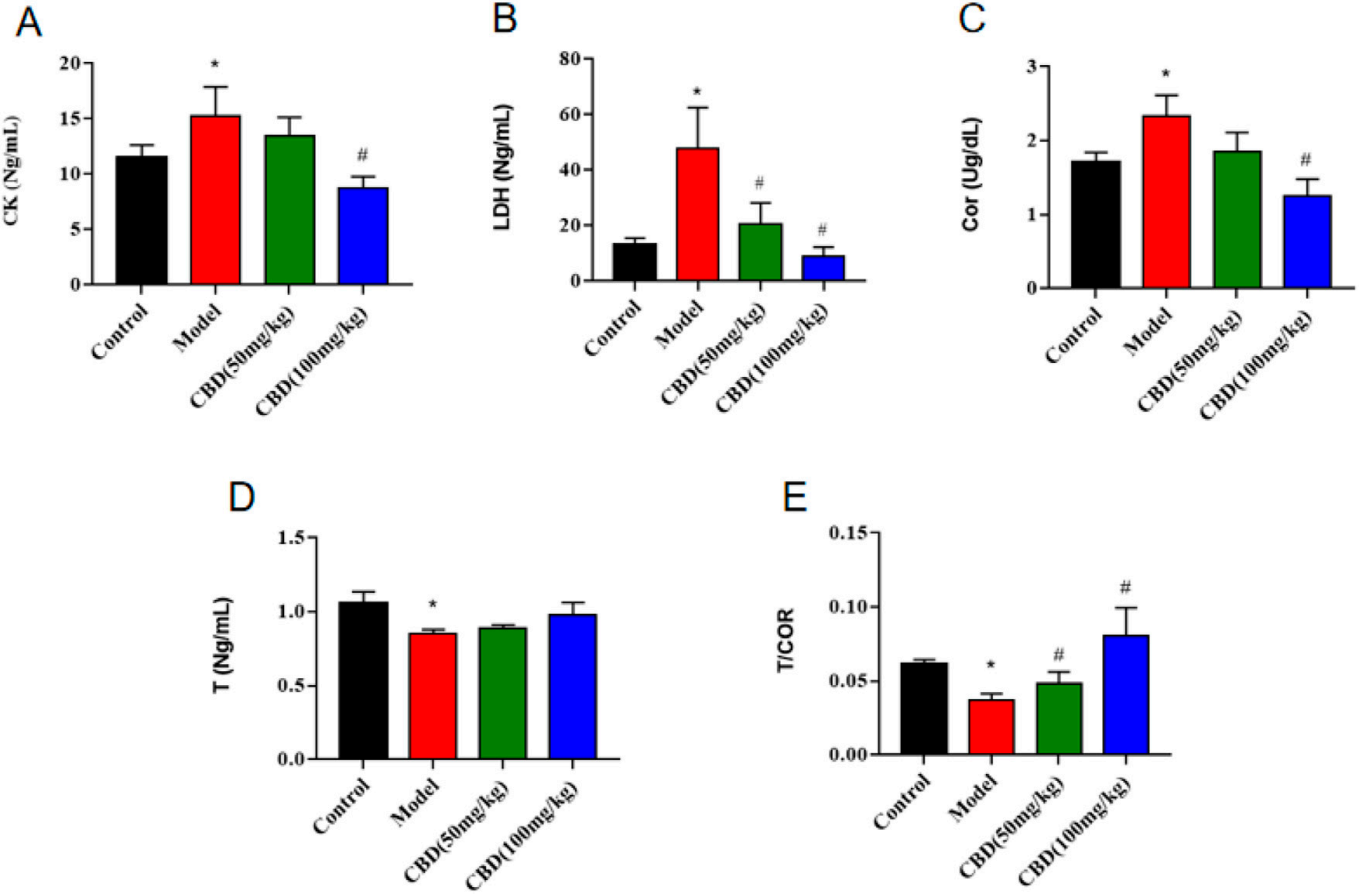
Effect of CBD on CK, T, Cor and LDH parameters in experimental rats. **(A)** CK (n = 6, one-way ANOVA). **(B)** LDH (n = 6, one-way ANOVA). **(C)** Cor (n = 6, one-way ANOVA). **(D)** T (n = 6, one-way ANOVA). **(E)** T/COR (n = 6, one-way ANOVA). All date are expressed as mean ± S.D. *p < 0.05 compared with the control group. ^#^ *p* < 0.05 compared with the model group.

As shown in Figure 2, compared with the control group, the content of serum Cor and the activities of CK and LDH in the model group were significantly increased (*p* < 0.05), the content of serum T and the ratio of T/Cor were significantly decreased (*p* < 0.05), and the mean value of T/Cor was 40.35% lower than that in the control group. Compared with the model group, the T/Cor ratio of rats in the high-dose CBD group was significantly increased (*p* < 0.05), the serum T content was increased, and the serum Cor content and the activities of CK and LDH were significantly decreased (*p* < 0.05). Compared with the model group, the activity of serum CK and the content of Cor in the low dose group of CBD were decreased, the activity of serum LDH was decreased (*p* < 0.05), the content of serum T was increased and the ratio of T/Cor was significantly increased (*p* < 0.05).

### 3.3 Effect of CBD on oxidative stress parameters in experimental rats

**FIGURE 3:**
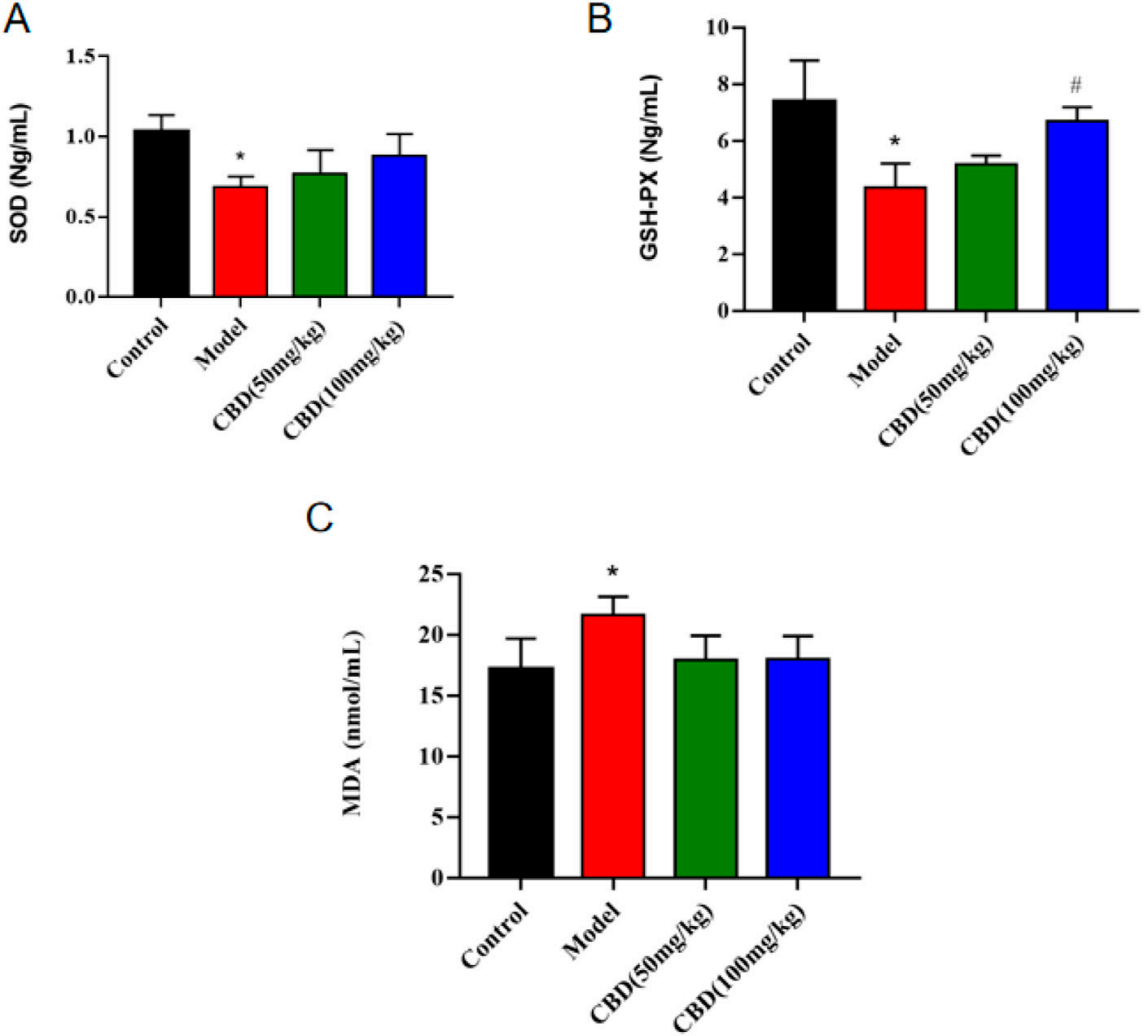
Effect of CBD on SOD, GSH-PX and MDA in experimental rats. **(A)** SOD (n = 6, one-way ANOVA). **(B)** GSH-PX (n = 6, one-way ANOVA). **(C)** MDA (n = 6, one-way ANOVA). All date are expressed as mean ± S.D. *p < 0.05 compared with the control group. ^#^ *p* < 0.05 compared with the model group.

As shown in Figure 3, compared with the control group, the activity of SOD and GSH-PX in serum of rats in the model group was significantly decreased (*p* < 0.05), and the content of MDA was significantly increased (*p* < 0.05). Compared with the model group, the activity of serum SOD and GSH-PX (*p* < 0.05) in the low dose group of CBD was increased, and the content of MDA was evidently decreased (*p* < 0.05). Compared with the model group, the serum SOD activity in the high-dose CBD group was obviously increased (*p* < 0.05), and serum GSH-PX activity was increased significantly (*p* < 0.05), and the serum MDA content was sensibly decreased (p < 0.05).

### 3.4 Effect of CBD on HE staining of gastrocnemius muscle in experimental rats

**FIGURE 4:**
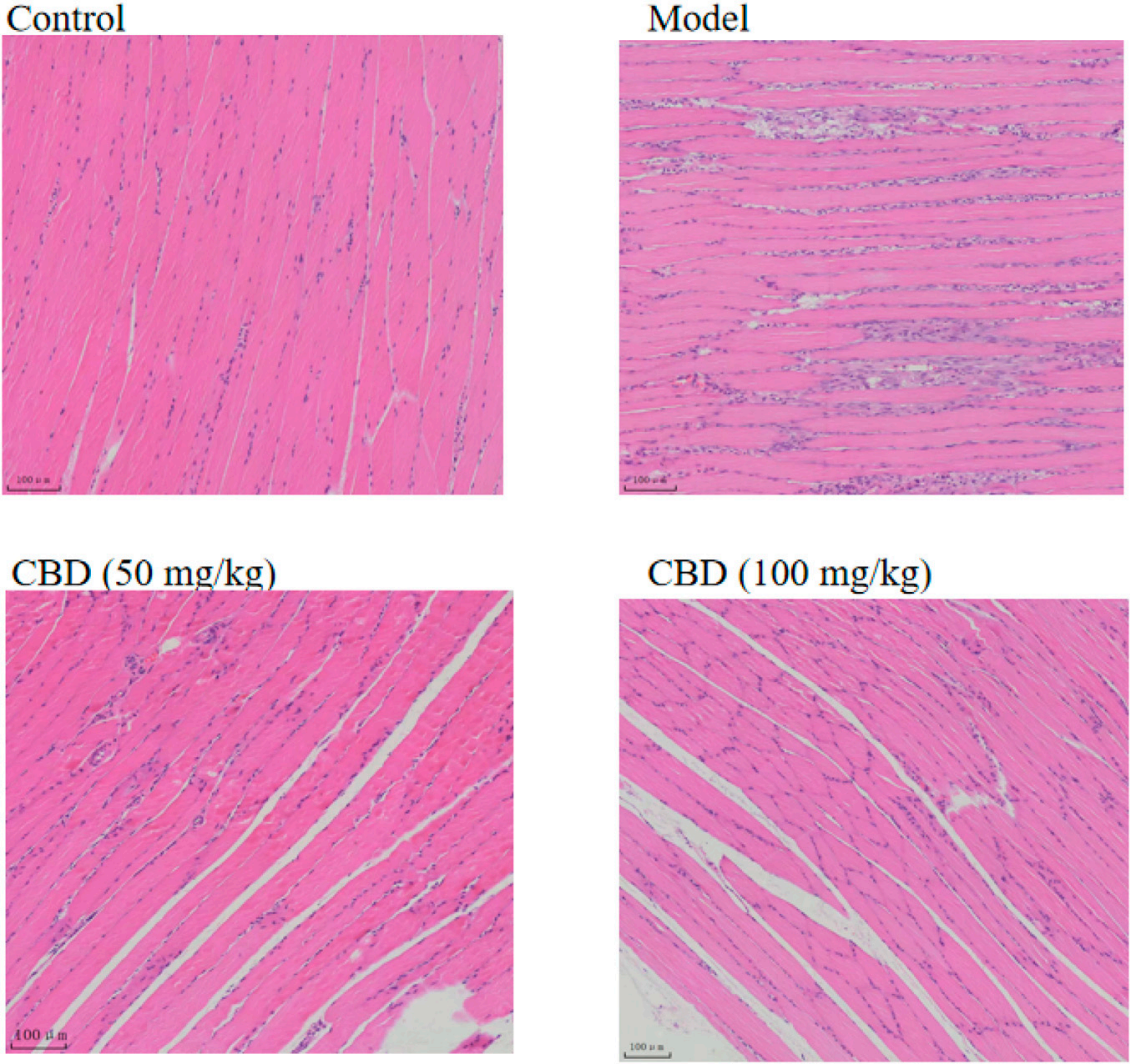
Effects of CBD on histopathology in the experimental rats. The skeletal muscle tissues were stained with H&E, and micrographs were taken at 100× magnification.

It can be seen from Figure 4 that the skeletal muscle morphology of rats in the control group was normal, and the shape and distribution of nucleus were normal. In the model group, the inflammatory cells of skeletal muscle were infiltrated seriously, the cells between muscle fibers proliferated, and some nuclei were displaced. The degree of skeletal muscle injury in the CBD dose group was lower than that in the model group, with occasional inflammatory cell infiltration and relatively normal nuclear distribution.

### 3.5 Transcriptome sequencing analysis

**FIGURE 5:**
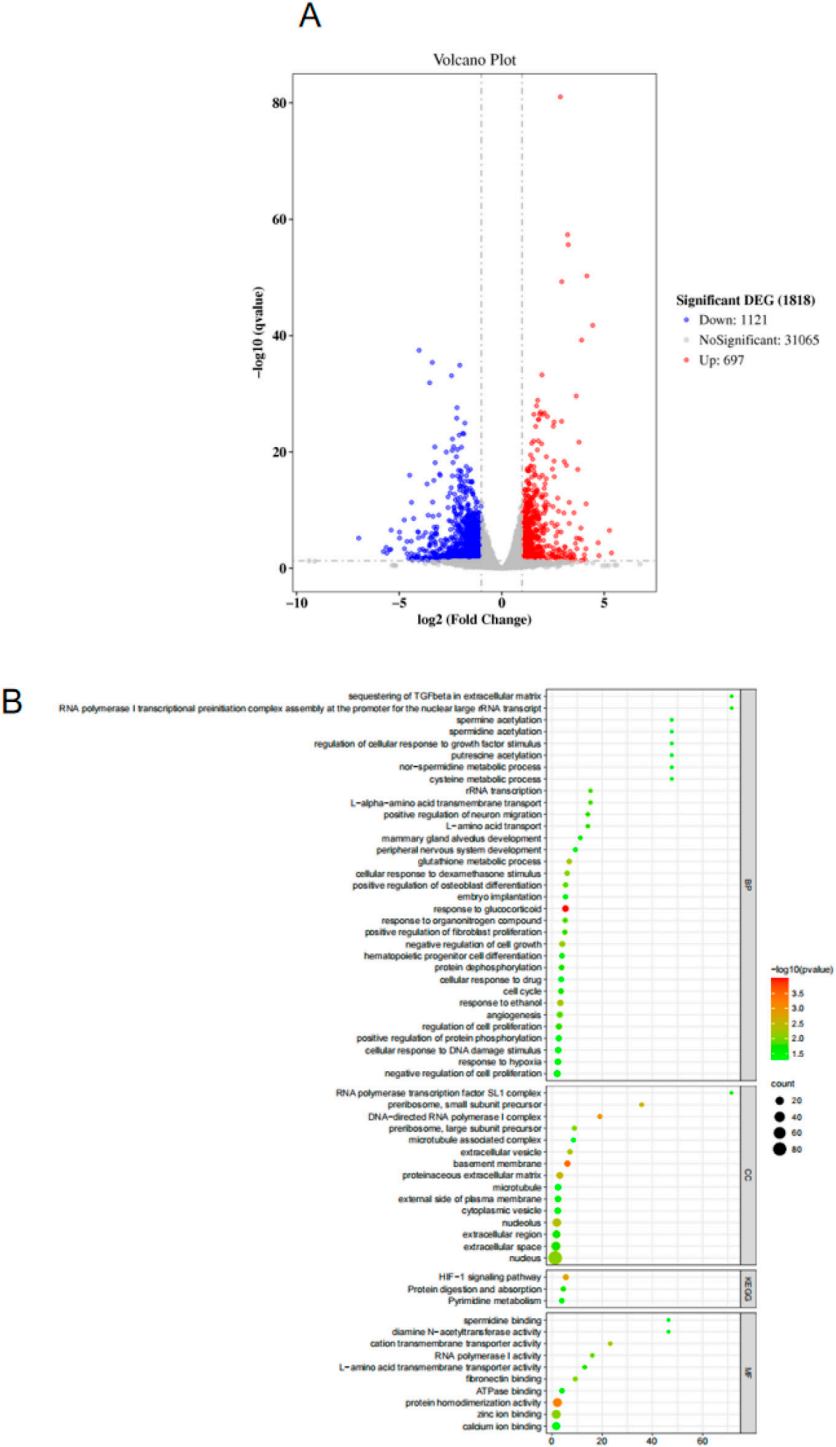
**(A)** Volcano map for screening differentially expressed genes. **(B)** GO and KEGG analysis results.

As shown in Figure 5A, there are 1818 DEGs between the control group and the model group.Compared with the model group, 697 genes were upregulated and 1121 genes were downregulated in the control group. As shown in Figure 5B, the GO function enrichment analysis was performed on the DEGs between the control group and the model group. There were 33 items related to BP, which involved sequesting of TGF-β in extracellular matrix, spermine acetylation, regulation of cellular response to growth factor stimulus, putrescine acetylation, nor-spermidine metabolic process, cysteine metabolic proces, etc. There were 15 CC-related items, involving RNA polymerase transcription factor SL1 complex, preribosome, small subunit precursor, DNA-directed RNA polymerase 1 complex, preribosome, large subunit precursor, microtubule associated complex A and B, etc. There were 10 MF-related items, involving spermidine binding, dlamine N-acetyltransferase activity, cation transmembrane transporter activity, RNA polymerase 1 activity, L-amino acid transmembrane transporter activity, etc.

Based on the KEGG database, enrichment analysis was conducted on the signaling pathways that differentially expressed genes may participate in. The results (Figure 5B) showed that the targets were mainly enriched in HiF-1 signaling pathway, Protein digestion and absorption and Pyrimidine metabolism, etc. The above signaling pathways may be involved in the role of CBD in skeletal muscle repair. The HiF-1 signaling pathway was selected in the following study to further analyze the potential mechanism of CBD in improving exercise-induced skeletal muscle injury.

### 3.6 Network analysis

**FIGURE 6:**
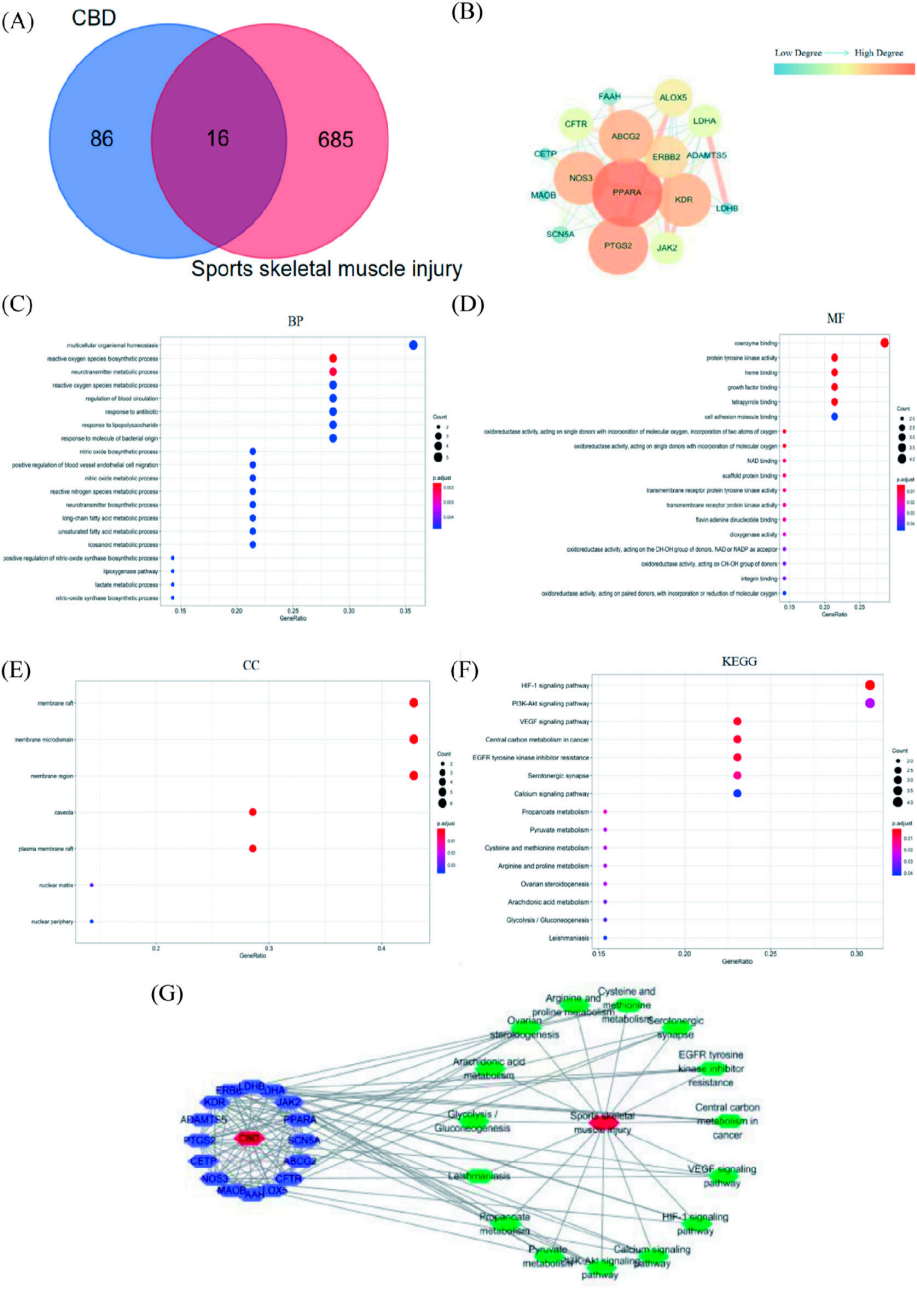
Results of network pharmacological analysis. **(A)**Venn Diagram of CBD targets and exercise-induced skeletal muscle injury targets. **(B)** PPI network of intersection targets of CBD in the treatment of exercise-induced skeletal muscle injury. **(C–E)** GO analysis bubble map of CBD. **(F)** KEGG analysis bubble map of CBD. **(G)** Network diagram of “component-target-pathway-disease.”

The smiles number of CBD retrieved from PubChem database is CCCCC 1 = CC (= C (c (=C1) O) C2C = C (CCC2C (=C) C) O. The Swiss Target Prediction database was used to retrieve 100 relevant targets of CBD. The GeneCards database was used to screen 635 targets related to sports skeletal muscle injury. The Venn diagram is plotted in Figure 6A. The targets of CBD were mapped to those of exercise-induced skeletal muscle injury, and 16 intersection targets were obtained (Supplementary Table S1).

The common targets of CBD and exercise-induced skeletal muscle injury were imported into String database for analysis, and Cytoscape software was used to beautify the protein interaction network (Figure 6B). There were 16 nodes and 66 edges in the network, the average node degree value was 8.25, and the average local clustering coefficient was 0.81. In this network, the degree value was selected as the screening basis, and the targets with degree value greater than the average node degree value were PPARA, PTGS2, ABCG2, NOS3, KDR, ERBB2 and ALOX5 in descending order of degree value.

As shown in Figures 6C–E, 212 GO enrichment entries were obtained (*p* < 0.05) after R language analysis. There were 18 entries related to molecular function (MF), involving coenzyme binding, protein tyrosine kinase activity and heme binding. There were 187 entries related to biological process (BP), involving reactive oxygen species biosynthetic process, neurotransmitter metabolic process, multicellular organismal homeostasis. There were 7 items related to cell component (CC), involving membrane raft and membrane microdomaint.

It can be seen from Figure 6F that after R language analysis, 15 enriched signal pathways were obtained (*p* < 0.05). The results showed that the targets were mainly enriched in HIF-1 signaling pathway, VEGF signaling pathway, Central carbon metabolism in cancer, EGFR tyrosine kinase inhibitor resistance. It was suggested that CBD may play a role in the treatment of exercise-induced skeletal muscle injury through the above signal pathways.

The path diagram of “component-target-path-disease” was shown in Figure 6G. There were 33 nodes and 135 edges in the network, including 16 purple nodes, which were common targets. There were 15 green nodes, which were the selected KEGG channels. There were 2 red nodes, which were respectively CBD and sports skeletal muscle injury. Among them, the degree values of common targets were ranked as PTGS2, NOS3, KDR, PPARA, LDHA, ERBB2, ALOX5, ABCG2 in descending order. From the largest to the smallest, the degrees of pathways were PI3K-Akt signaling pathway, HIF-1 signaling pathway, VEGF signaling pathway, EGFR tyrosine kinase inhibitor resistance, Serotonergic synapse, Calcium signaling pathway, Central carbon metabolism in cancer.

### 3.7 Effect of CBD on mRNA expression in skeletal muscle of experimental rats

**FIGURE 7:**
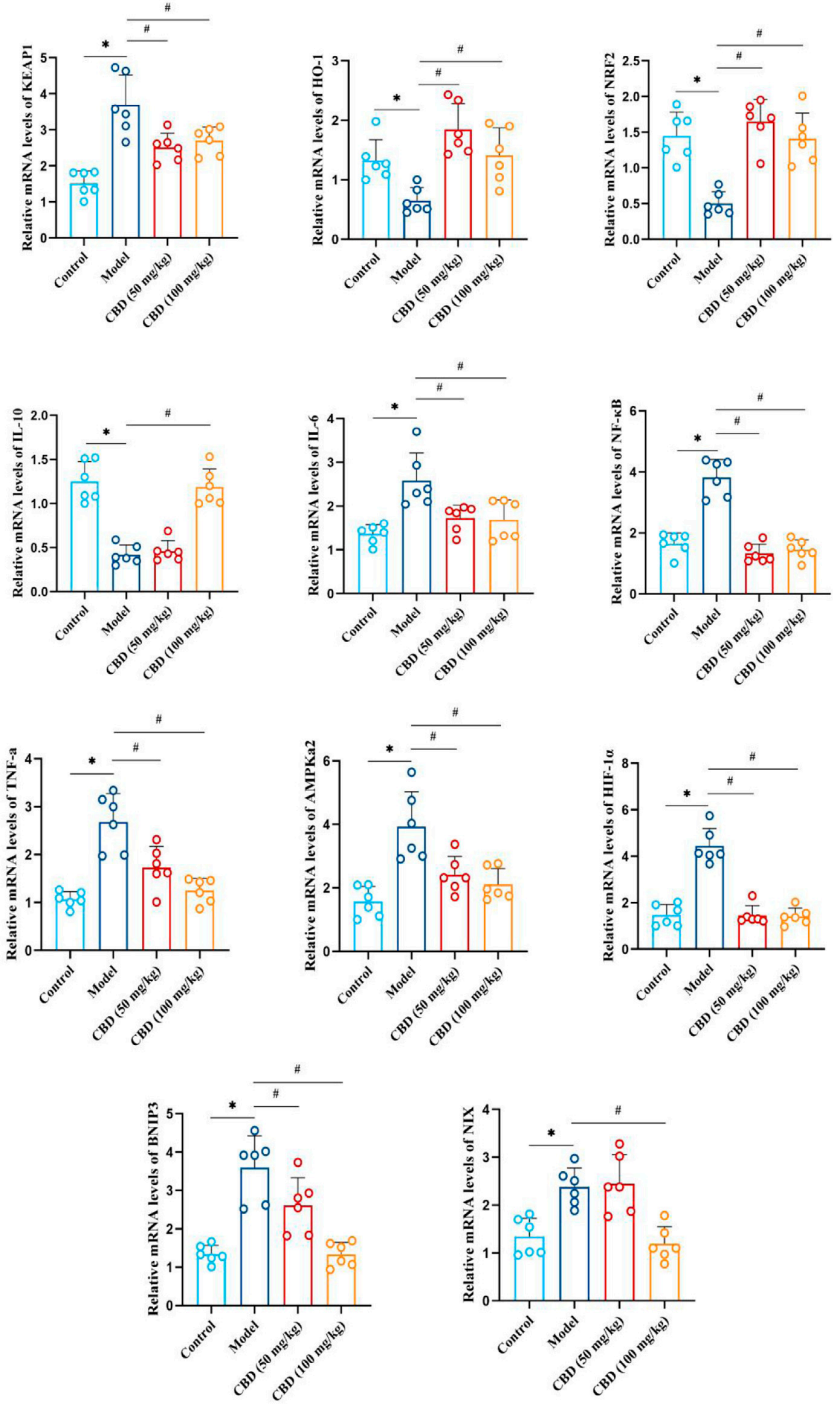
Effect of CBD on mRNA expression of HO-1, Nrf2, Keap1, IL-10, IL-6, NF-κB, TNF-α, AMPKα2, Hif-1α, BNIP3 and NIX in skeletal muscle of exhaustive exercise rats. Data are expressed as means ± S.D, one-way ANOVA, *p < 0.05 as compared to the control group; ^#^ *p* < 0.05 as compared to the model group.

As indicated in Figure 7, compared with the control group, the mRNA expression of Keap1, IL-6, NF-κB, TNF-α, AMPKα2, HIF-1α, BNIP3, and NIX in the skeletal muscle tissue of the model group was sensibly increased (*p* < 0.01). The mRNA expression of HO-1, Nrf2 and IL-10 was markedly decreased compared to the control group (*p* < 0.01). Compared with that the model group, the mRNA expression of Keap1, IL-6, NF-κB, TNF-α, AMPKα2, HIF-1α, BNIP3 and NIX in the skeletal muscles of rats in the low-dose and high-dose CBD groups was evidently reduced (*p* < 0.01), while HO-1, Nrf2 and IL-10 were obviously increased (*p* < 0.01). Among them, the improvement effect of CBD high dose group was better.

### 3.8 Effect of CBD on protein expression of gastrocnemius muscle in experimental rats

**FIGURE 8:**
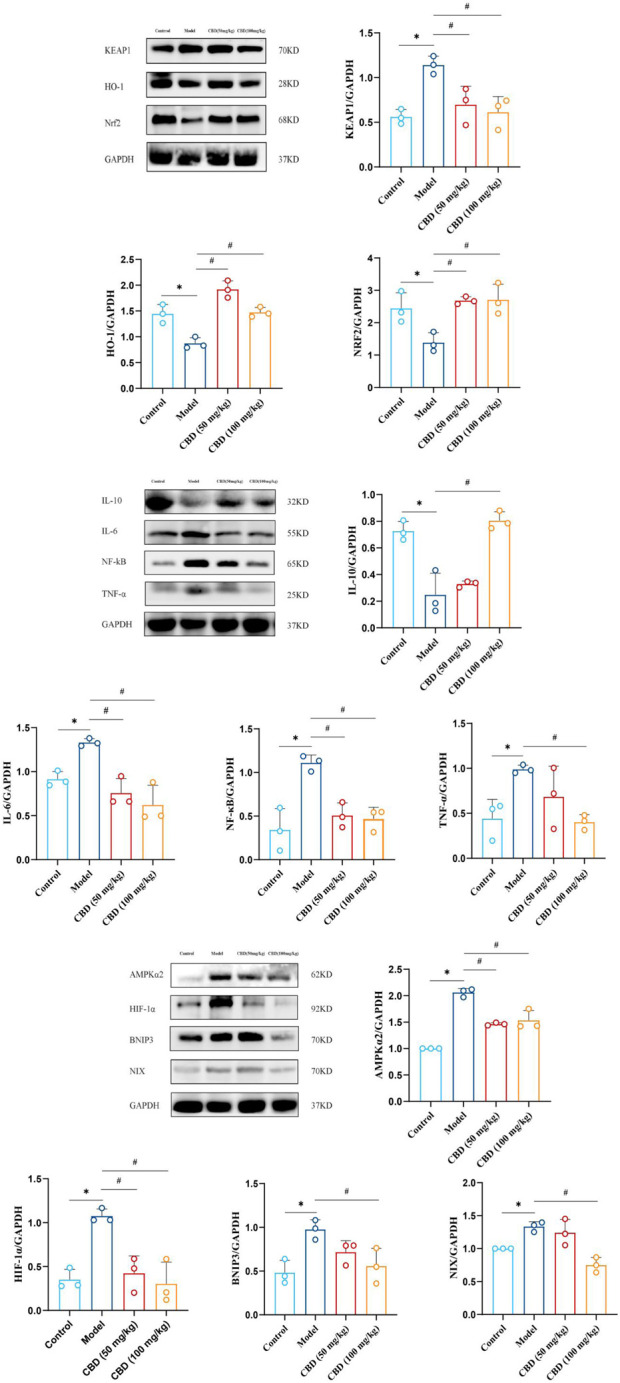
The analysis of western blot for CBD in skeletal muscle of experimental rats and quantification of protein expression. The protein levels (HO-1, Nrf2, Keap1, IL-10, IL-6, NF-κB, TNF-α, AMPKα2, Hif-1α, BNIP3, NIX) were normalized to GAPDH. Data are expressed as means ± S.D of triplicate experiments performed independently, one-way ANOVA, *p < 0.05 as compared to the control group; ^#^ *p* < 0.05 as compared to the model group.

As shown in Figure 8, compared with the control group, the expression of HO-1 and Nrf2 protein in skeletal muscle of rats in the model group was significantly decreased (*p* < 0.05), and the expression of Keap1 was significantly increased (*p* < 0.05). Compared with the model group, the expression of HO-1 and Nrf2 protein in skeletal muscle of rats in the low-dose and high-dose CBD groups was increased significantly (*p* < 0.05), while the expression of Keap1 protein was decreased significantly (*p* < 0.05).

It can be seen from Figure 8 that compared with the control group, the expressions of NF-kB, TNF-α and IL-6 in skeletal muscle of the model group were significantly increased (*p* < 0.05), while the expression of IL-10 was significantly decreased (*p* < 0.05). Compared with the model group, the expressions of NF-kB and IL-6 in skeletal muscle of the low-dose and high-dose CBD groups were significantly decreased (*p* < 0.05). In the low-dose CBD group, the expression of TNF-α was decreased, and the expression of IL-10 was increased. The expression of TNF-α in the high-dose CBD group was significantly decreased (*p* < 0.05), and the expression of IL-10 was significantly increased (*p* < 0.05).

As shown in Figure 8, compared with the control group, protein expressions of AMPKα2, Hif-1α, BNIP3 and NIX in skeletal muscle of the model group were significantly increased (*p* < 0.05). Compared with the model group, the expressions of AMPKα2 and Hif-1α in skeletal muscle of the low-dose and high-dose CBD groups were significantly decreased (*p* < 0.05). The expressions of BNIP3 and NIX were decreased in the low dose CBD group comparing with the model group. The expressions of BNIP3 and NIX in the high-dose CBD group were significantly decreased (*p* < 0.05) by comparison of the model group.

## 4 Discussion

The primary purpose of this study was to accurately establish the animal model of sports injury. Based on this, an improved Bedford incremental load exercise model was used to establish the model group, the control group, and the low-dose and high-dose groups of CBD. In order to obtain the action targets and related pathways of CBD in the treatment of exercise-induced skeletal muscle injury, transcriptome analysis and network analysis were performed. The aim is to confirm the theory from the molecular level and further elucidate the mechanism of action between the active ingredient and the target.

It was reported that the activities of serum CK and LDH were sensitive indicators to evaluate skeletal muscle injury, and the activities of serum CK and LDH were positively correlated with the degree of skeletal muscle mechanical injury (Li, et al., 2023; Brancaccio et al., 2006; Nie, et al., 2011). The results of this study showed that compared with the control group, the activities of serum CK and LDH in the model group were significantly increased (*p* < 0.05). The activities of serum CK and LDH in the low-dose and high-dose groups of CBD were decreased comparison with the model group. The serum T/Cor ratio is the gold standard for evaluating exercise load and diagnosing overtraining (Wei et al., 2023). The experimental results indicated that compared with the control group, the T/Cor ratio in the serum of the model group was decreased significantly, reaching the relevant diagnostic criteria for overtraining. The intervention of CBD could increase the T/COR ratio. At the same time, the HE staining pathological section also showed that the skeletal muscle in the model group had abnormal arrangement of muscle fibers, segmental fracture injury, dissolution and even disappearance, and other pathological phenomena.

Long-term strenuous exercise can lead to imbalance between oxidation and antioxidant system, which will increase the oxidative stress of the body. Oxidative stress caused by exercise may be one of the important reasons for exercise-induced skeletal muscle injury (Smith, et al., 2023; Zhang et al., 2022). MDA, the product of lipid peroxidation, can reflect the degree of lipid peroxidation and indirectly reflect the degree of cell membrane damage (Zuo, et al., 2023; Forman and Zhang, 2021). SOD, as the main antioxidant enzyme of the body, can reflect the ability of scavenging free radicals (Batinić-Haberle et al., 2009). GSH-Px is also an intracellular antioxidant enzyme that can prevent oxidative stress (Wang et al., 2023). The results indicated that the content of MDA in the serum of the model group was significantly increased after exercise (*p* < 0.05), and the activities of SOD and GSH-PX were decreased significantly (*p* < 0.05), suggesting that acute vigorous exercise resulted in a large amount of free radicals in the skeletal muscle of rats, increased lipid peroxidation reaction, and then caused the structural damage and functional decline of muscle cell membrane. The content of MDA in the serum of rats in the low-dose and high-dose groups of CBD was lower than that in the model group, and the activities of SOD and GSH-PX were higher than those in the model group, suggesting that CBD supplementation could reduce lipid peroxidation, improve the body’s antioxidant capacity, and reduce the degree of exercise-induced skeletal muscle micro-damage.

More than 90% of antioxidant genes are regulated by Nrf2, and the regulation is mainly conducted at the post-translation level (Tonelli, 2018; Li et al., 2022). As an important antioxidant regulator, Nrf2 combines with Kelch sample-related protein-1 (Keap1) in the cytoplasm to form a complex. After the occurrence of oxidative stress, the complex of Nrf2 and Keapl is dissociated, translocated into the nucleus, and combined with the downstream gene heme monooxygenase-1 (HO-1) to play an antioxidant role (Liu and Malik, 2006). This study found that compared with the control group, the antioxidant capacity of skeletal muscle tissue in model group decreased under oxidative stress. Compared with the model group, the expression of anti-oxidative stress genes and proteins in skeletal muscle tissues of all drug administration groups was increased, especially in high-dose CBD group. These results suggest that CBD may be related to the regulation of Nrf2, Keap1 and HO-1 expression. It was consistent with our previous discovery that the CBD can regulate Nrf2/HO-1 signaling pathway (Zhang et al., 2022).

Moreover, skeletal muscle injury can quickly cause inflammatory reaction, and activated macrophages induced by muscle injury can quickly trigger excessive production of inflammatory factors such as IL-6, IL-10 and TNF-α (Wang et al., 2014). Inflammatory response can activate NF-κB, which plays a key role in inflammatory response and controls the production of inflammatory mediators, including IL-6, IL-10 and TNF-α (Hansson and Libby, 2006; Hu et al., 2020). This study suggested that CBD pre-protection could reduce the mRNA and protein expression of IL-6, TNF-α and NF-κB, and the mRNA and protein expression of IL-10 were increased. Studies have reported that inflammation promotes the inflammatory immune response of the body, produces excessive immune complex, activates complement, produces chemokines to attract inflammatory cells, releases ROS, and then causes oxidative stress response (Soberanes et al., 2009). Studies have pointed out that excessive ROS may also stimulate NF-κB, a key molecule in the inflammatory pathway, and cause repeated cell and tissue damage (Hardy and Rogaeva, 2014; Yu et al., 2020). The activation of NF-κB can be inhibited correspondingly when antioxidants are used (Hardy and Rogaeva, 2014; Yu et al., 2020). Therefore, oxidative stress and inflammation can regulate each other.

Based on network analysis and transcriptomics, this study explored the role of CBD in the repair of skeletal muscle injury caused by exhaustive exercise, which was closely related to HIF-1 signal pathway. Therefore, the signal path was deeply explored in the subsequent experiments. The HIF-1 transcription factor is an important regulator in hypoxic-induced response, which is sensitive to oxygen α Subunit (HIF-1 α) Then, the constitutive expression β Subunit (HIF-1β) Composition. Studies have shown that AMPK can regulate HIF-1α to promote hypoxia adaptation in the body and cells, and the expression of AMPK and HIF-1α may be synchronous after blunt trauma (Han et al., 2021; Sun et al., 2021). More and more studies have shown that oxidative stress can also directly or indirectly activate AMPK through ROS or RNS (Barone et al., 2021; Hartwig et al., 2021). The mammalian AMPK subunit has 7 genes, including 2 α subunits (α1 and α2), 2 β subunits (β1 and β2) and 3 γ subunits (γ 1,γ 2 and γ 3) (Lee-Young et al., 2010; Niu et al., 2017). AMPKα2 is mainly distributed in the nucleus of skeletal muscle (Fu et al., 2020; Sulkshane et al., 2021). This result was consistent with this experimental study. BNIP3 and NIX are the response genes of HIF-1α (Cui et al., 2022; He et al., 2017). BNIP3 and NIX are a class of genes located in mitochondrial outer membrane, which are expressed by hypoxia induction and participate in mitochondrial autophagy process (Cui et al., 2022; He et al., 2017; You et al., 2021). In this study, compared to the control group, the mRNA and protein expressions of BNIP3 and NIX were increased in the model group, which was consistent with the change of HIF-1α expression, indicating that exhaustive exercise may cause skeletal muscle injury. After the intervention of CBD, the expression levels of these factors were significantly regulated.

## 5 Conclusion

This study preliminarily explored the protective effect of CBD on skeletal muscle in the rat model of acute exercise-induced skeletal muscle injury. The CBD intervention can reduce CK and LDH levels and increase T/COR ratio. The MDA content in the low-dose and high-dose groups of CBD was declined, while the SOD and GSH-Px content were raised. The intervention of CBD can reduce the level of oxidative stress and inflammatory response, and then reduce the expressions of AMPKα2, HIF-1α, BNIP3 and NIX, thus protecting skeletal muscle from injury. This study could provide a new potential target for the treatment of exercise-induced skeletal muscle injury. It can provide new ideas for the basic research and clinical treatment of CBD repairing skeletal muscle injury in the future.

## Data Availability

The raw data has been deposited at the NCBI repository (BioProject), accession number PRJNA1160342. Available at: https://www.ncbi.nlm.nih.gov/bioproject/PRJNA1160342.
