# Supplementary material for: Effects of cannabidiol on AMPKα2 /HIF-1α/BNIP3/NIX signaling pathway in skeletal muscle injury
Source: Front Pharmacol. 2024 Oct 22;15:1450513. doi: 10.3389/fphar.2024.1450513 (PMC11536269; doi:10.3389/fphar.2024.1450513)
Supplement: Supplementary file 1 [file DataSheet1.PDF]

Tab. S1. Common target information of CBD

No.	Symbol
1	KDR
2	ABCG2
3	PPARA
4	ALOX5
5	MAOB
6	ERBB2
7	LDHB
8	NOS3
9	CFTR
10	CETP
11	FAAH
12	LDHA
13	JAK2
14	PTGS2
15	ADAMTS5
16	SCN5A
